# A Singular Case

**Published:** 1872-12

**Authors:** J. N. Robinson


### A SINGULAR CASE.

Reported by J. N. ROBINSON, M. D.

Mr. Andrew Haight, of Sharon, Medina county, Ohio, had been complaining for some time, yet able to go about.

On Sunday, June 9th, he was taken with severe pain in his side and bowels, which was partially relieved by remedies, yet the general symptoms appeared more and more aggravated, and a *stercoraceous* vomiting ensued.

Mr. H. had formerly been troubled with umbilical and inguinal hernia, but neither was detected by the attending physician (homœopathy), and it neither proved to be strangulated or irreducible.

The friends and physician became alarmed at the steady progress and fatal appearance of the disease, and summoned counsel, but, before it arrived, death ensued—Thursday, June 13th—with but little increase of pain and a very obscure diagnosis.

On the arrival of the counsel, several earnest and inquiring friends assembled determined to know the cause of his premature death. And about four hours after, a *post mortem* was held, which developed obstruction in the ilium, composed of a concrete substance of *cholesterin* and feculent matter, very symmetrical in form, and hard, about one inch in diameter and two inches long, completely filling the ilium, and spasmodically grasped by it about two feet above the ileo-cæcal valve.

The examination showed extensive enteritis, not only above, but below the obstruction, and no more where the substance was lodged than at other points. and the mucus membrane was as perfect there as anywhere above, no more inflamed or irritated ; and there was no gangrene or breaking down of structure. The examination of this concrete substance shows no nucleus of magnesia, chalk, or gall-stone.

